# Supplementary material for: Selective Destabilization of Transcripts by mRNA Decapping Regulates Oocyte Maturation and Innate Immunity Gene Expression during Ageing in C. elegans
Source: Biology (Basel). 2023 Jan 21;12(2):171. doi: 10.3390/biology12020171 (PMC9952881; doi:10.3390/biology12020171)
Supplement: Supplementary file 1 [file biology-12-00171-s001.zip › Supplem Figures.pdf]

Supplementary Figure legends

Figure S1. Downstream analysis of microarray data. (A) Correlation of microarray results with qRT-PCR quantification in the same (red line) or different (blue line) set of samples (Pearson correlation analysis). (B) Oogenic/spermatogenic classification of germline-specific transcripts that are differentially expressed in *dcap-1(rf)* animals. Datasets of germline-specific, oogenic and spermatogenic transcripts were determined as described in the main text. See also Table S4.

Figure S2. Relative fluorescence of wild type and *dcap-1(rf)* worms expressing the *irg-5p::gfp* transcriptional reporter at various ages. Symbols represent individual values. Bars represent mean ± SEM. All values are normalized to the average fluorescence of 1 day old wild type animals. **p*≤0.05, ***p*≤0.01, ****p*≤0.001, *****p*≤0.0001. Unpaired *t*- test.

Figure S3. Reduced function of DCAP-1 facilitates the translocation of PQM-1 to nucleus. Representative confocal fluorescent images (maximum intensity Z projections) of wild type and *dcap-1(rf)* animals expressing a *pqm-1::gfp* translational reporter under the control of the native *pqm-1* promoter, at various ages. Scale bar=50μm.

Figure S4. Reduced function of DCAP-1 delays the aggregation of Q35 peptides during ageing. (A) Representative fluorescent images (maximum intensity Z projections) of the head region of worms expressing Q35::YFP in their muscles at various ages. Scale bar=20μm. (B) Number of Q35 aggregates per worm at various ages. Symbols represent individual values, bars represent mean ± SD. **p*≤0.05, ***p*≤0.01, ****p*≤0.001, *****p*≤0.0001. One-way Anova with Sidak's correction.

Figure S5. Reduced function of DCAP-1 does not increase the stability of *eft-3* mRNA. (A) Relative levels of mature ($mRNA_{mat}$) and primary ($mRNA_{pri}$) *eft-3* transcripts in wt and *dcap-1(rf)* worms at the 1st and the 9th day of adulthood. (B) Stability of mature *eft-3* transcripts in wt and *dcap-1(rf)* worms at the 1st and the 9th day of adulthood. **p*<0.05. Unpaired *t*- test.

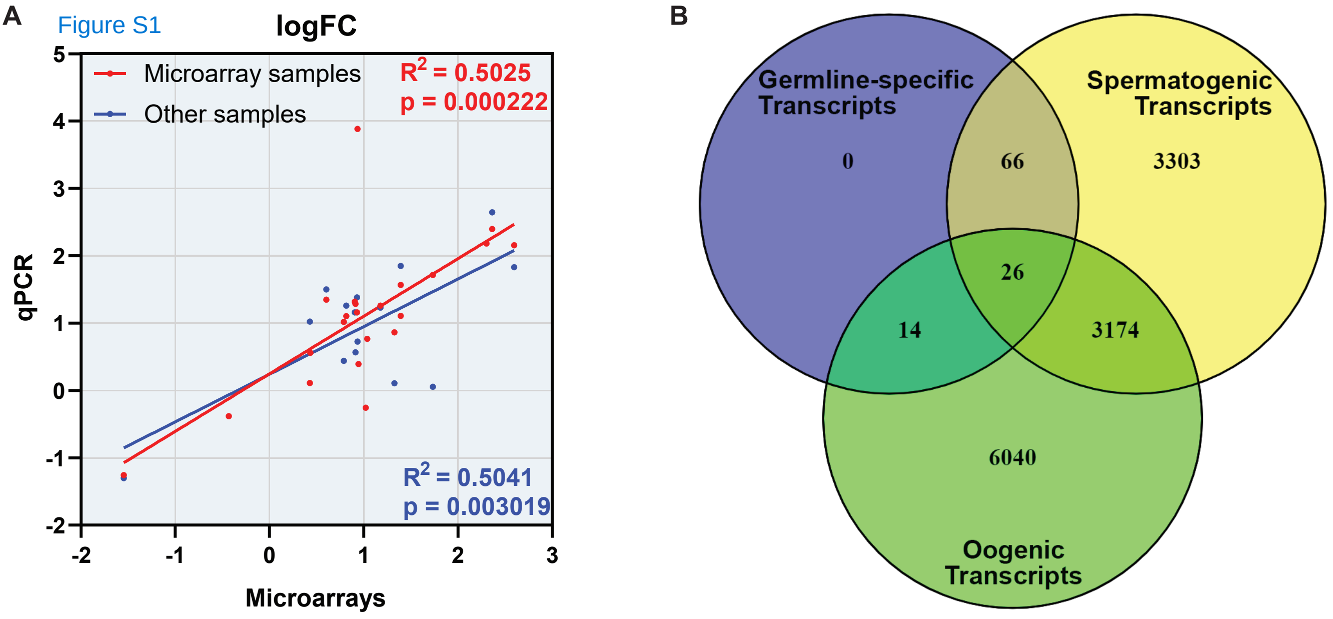

A
Figure S1
logFC
Microarray samples
Other samples
R2 = 0.5025
p = 0.000222
R2 = 0.5041
p = 0.003019
qPCR
Microarrays
5
4
3
2
1
0
-1
-2
-2
-1
0
1
2
3
B
Germline-specific Transcripts
Spermatogenic Transcripts
Oogenic Transcripts
0
66
3303
26
14
3174
6040

Figure S2

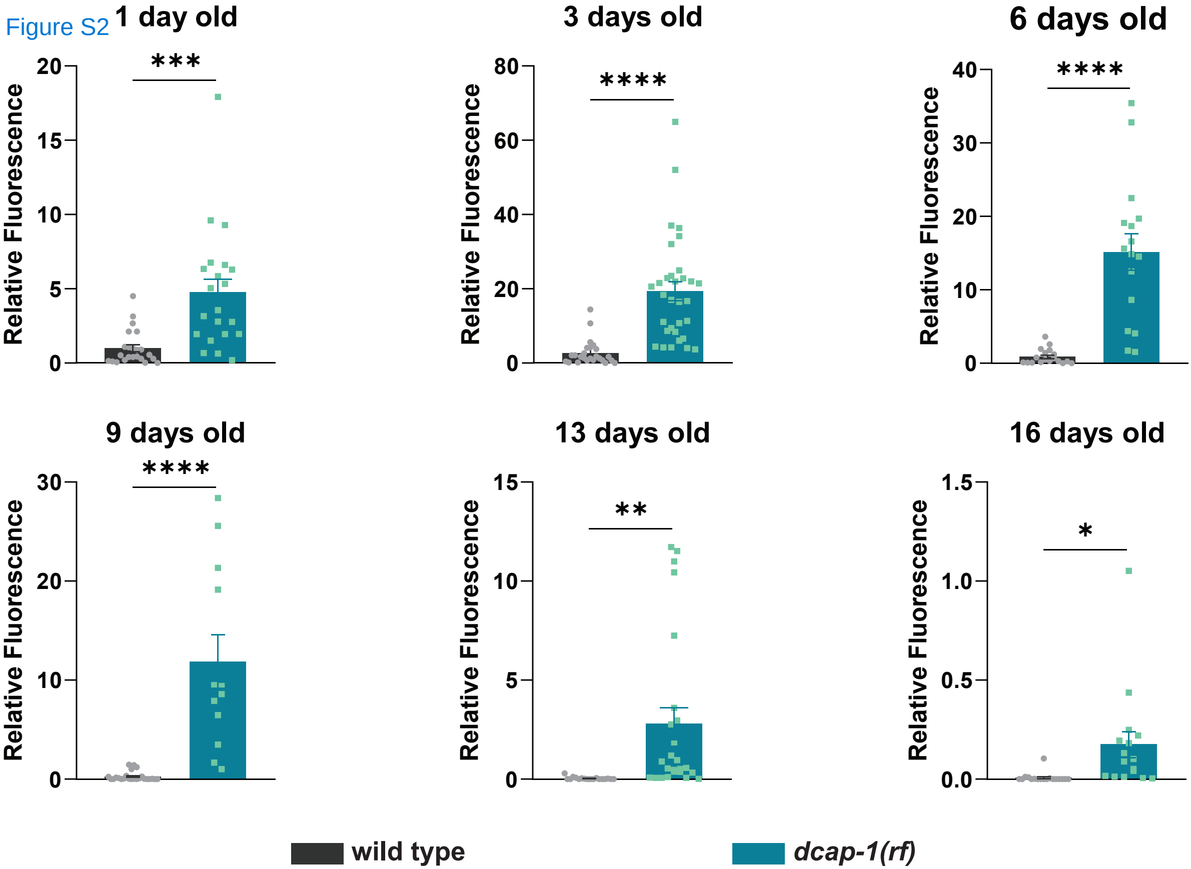

1 day old
3 days old
6 days old
9 days old
13 days old
16 days old
Relative Fluorescence
***
****
****
****
**
*
20
15
10
5
0
80
60
40
20
0
40
30
20
10
0
30
20
10
0
15
10
5
0
1.5
1.0
0.5
0.0
wild type
dcap-1(rf)

Figure S3

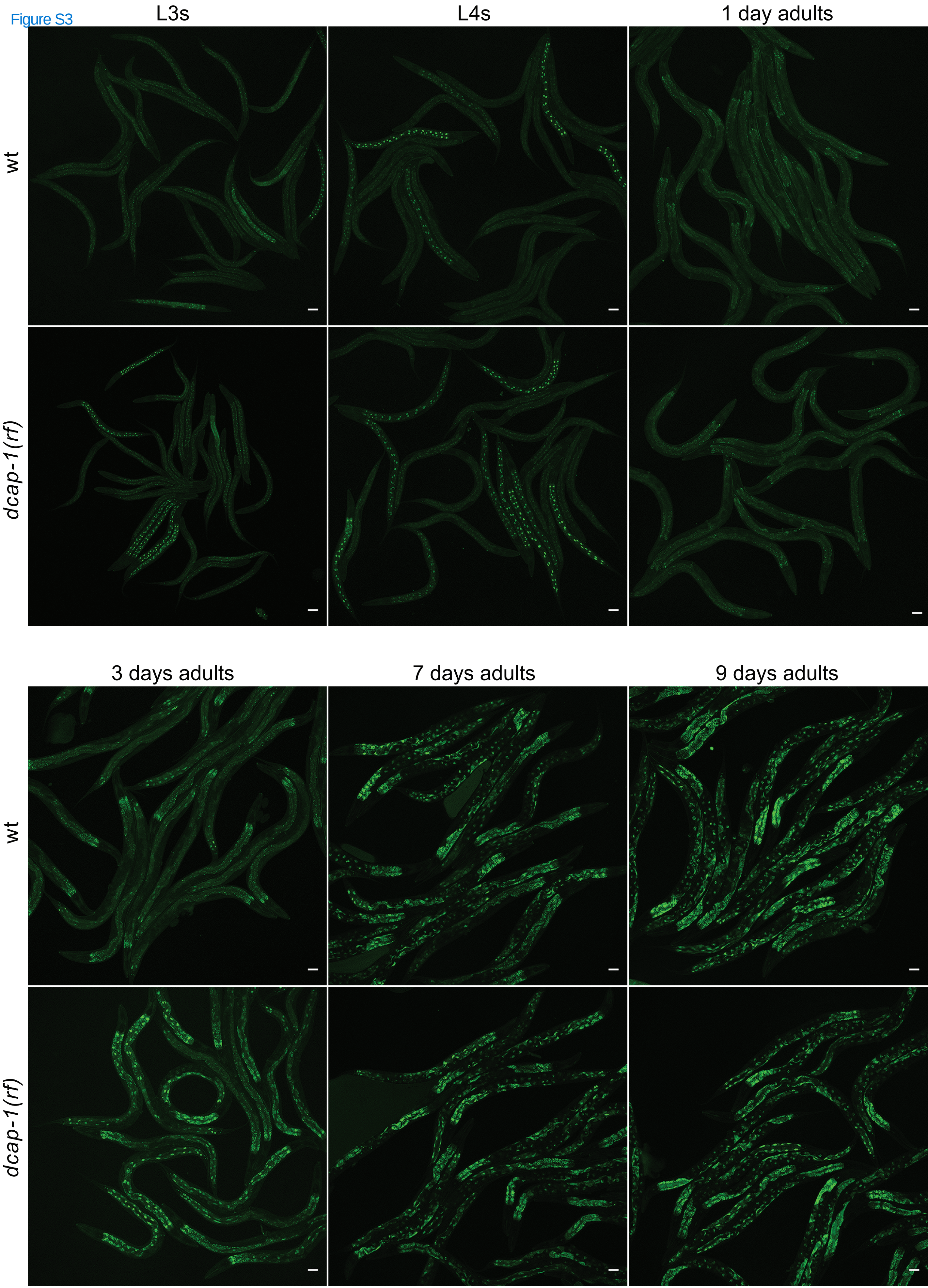

L3s
L4s
1 day adults
wt
dcap-1(rf)
3 days adults
7 days adults
9 days adults
wt
dcap-1(rf)

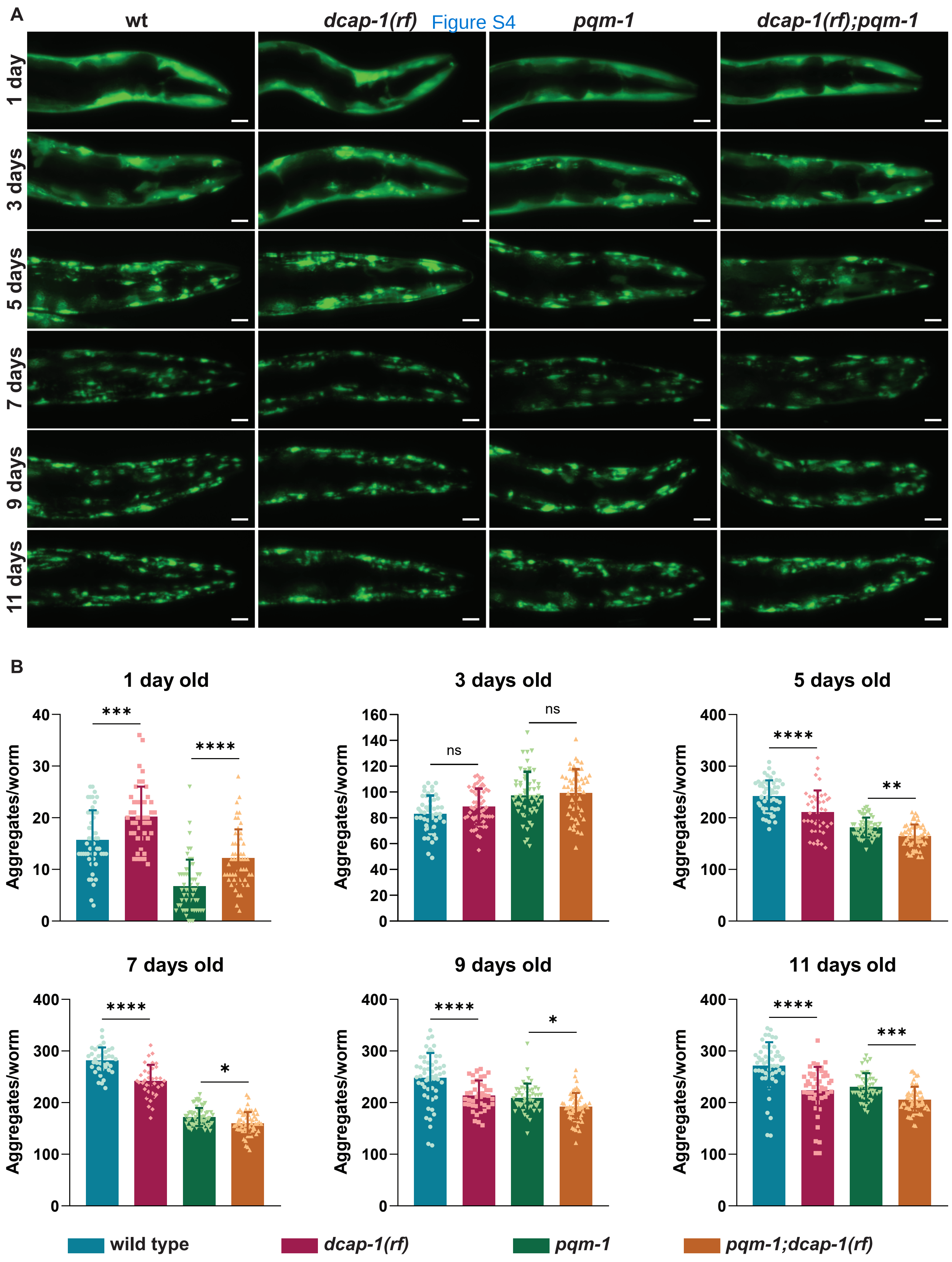
Figure S4
A
wt
dcap-1(rf)
pqm-1
dcap-1(rf);pqm-1
1 day
3 days
5 days
7 days
9 days
11 days
B
1 day old
3 days old
5 days old
7 days old
9 days old
11 days old
Aggregates/worm
***
****
ns
**
*
wild type
dcap-1(rf)
pqm-1
pqm-1;dcap-1(rf)

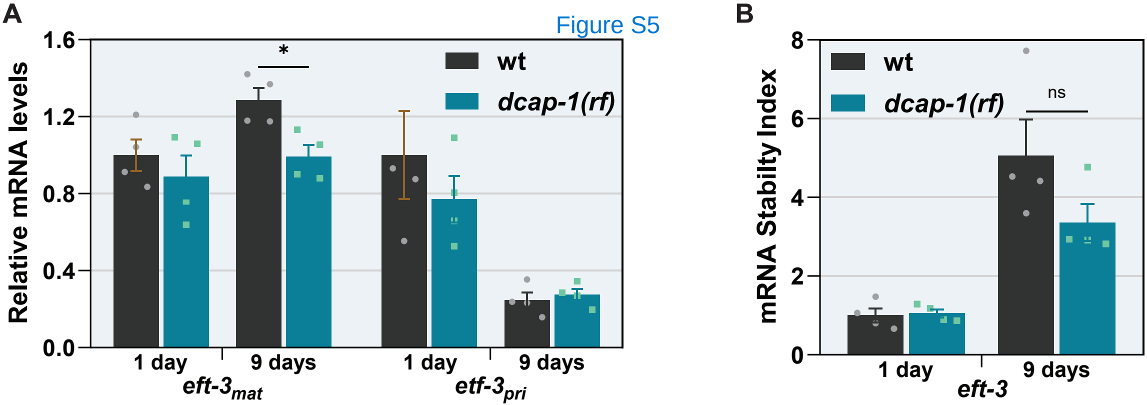

A
Figure S5
B
1.6
1.2
0.8
0.4
0.0
Relative mRNA levels
wt
dcap-1(rf)
*
1 day
9 days
1 day
9 days
eft-3$_{mat}$
etf-3$_{pri}$
8
6
4
2
0
mRNA Stabilty Index
ns
eft-3
